# A Novel Method for Rolling Bearing Fault Diagnosis Based on Gramian Angular Field and CNN-ViT

**DOI:** 10.3390/s24123967

**Published:** 2024-06-19

**Authors:** Zijun Zhou, Qingsong Ai, Ping Lou, Jianmin Hu, Junwei Yan

**Affiliations:** 1 School of Information, Wuhan University of Technology, Wuhan 430070, China; zijunzhou@whut.edu.cn (Z.Z.); qingsongai@whut.edu.cn (Q.A.); louping@whut.edu.cn (P.L.); 2 School of Information Engineering, Hubei University of Economics, Wuhan 430205, China; hujianmin@hbue.edu.cn

**Keywords:** fault diagnosis, gramian angular field, convolutional neural network, vision transformer, sensors

## Abstract

Fault diagnosis is one of the important applications of edge computing in the Industrial Internet of Things (IIoT). To address the issue that traditional fault diagnosis methods often struggle to effectively extract fault features, this paper proposes a novel rolling bearing fault diagnosis method that integrates Gramian Angular Field (GAF), Convolutional Neural Network (CNN), and Vision Transformer (ViT). First, GAF is used to convert one-dimensional vibration signals from sensors into two-dimensional images, effectively retaining the fault features of the vibration signal. Then, the CNN branch is used to extract the local features of the image, which are combined with the global features extracted by the ViT branch to diagnose the bearing fault. The effectiveness of this method is validated with two datasets. Experimental results show that the proposed method achieves average accuracies of 99.79% and 99.63% on the CWRU and XJTU-SY rolling bearing fault datasets, respectively. Compared with several widely used fault diagnosis methods, the proposed method achieves higher accuracy for different fault classifications, providing reliable technical support for performing complex fault diagnosis on edge devices.

## 1. Introduction

With the advancement of Industry 4.0 and intelligent manufacturing, the Industrial Internet of Things (IIoT) is rapidly becoming a core component of modern industrial systems. Edge computing, by placing computing resources and data storage near the data generation source at edge nodes, greatly reduces data transmission latency and bandwidth requirements, providing a more efficient solution for data processing and analysis. Fault diagnosis is one of the important applications of edge computing in the industrial IoT. In the industrial IoT environment, devices and sensors are interconnected via networks, and data analysis and fault diagnosis are performed at the device end, enabling the system to respond to anomalies more quickly. However, fault diagnosis methods face greater challenges in this context. First, there is the issue of high latency and bandwidth limitations in data transmission. In traditional centralized data processing systems, all data must be transmitted to a central server for processing and analysis. This approach is impractical in an IIoT environment due to the enormous volume of data generated by industrial devices and sensors, which can cause network congestion, increase latency, and reduce the system’s response speed. Secondly, edge nodes have limited resources. Edge computing devices usually have lower computing power and storage capacity compared to cloud servers, making resources relatively scarce. Furthermore, the heterogeneity of IIoT devices and the dynamic nature of the environment add to the difficulty of fault diagnosis. IIoT systems encompass various types of devices and sensors, each with different operating conditions, working modes, and fault types. Therefore, researching efficient fault diagnosis methods for mechanical equipment is of significant importance for improving the operational efficiency and lifespan of individual devices and ensuring the stable operation and continuous development of industrial systems.

Rolling bearings are critical components in rotating machinery, and their health, such as cracks or faults in different parts, directly affects the performance, efficiency, stability, and lifespan of the equipment [1]. As industry develops, most rotating machinery needs to withstand enormous workloads. When rolling bearings operate under high loads, strong impacts, heavy workloads, and complex environments, faults often occur in the inner ring, outer ring, balls, and cage. These faults cause the characteristics of the vibration sensor signals to differ from the normal vibration patterns, and fault information is embedded in these vibration signals. If these faults are not detected and addressed promptly, the equipment often stops working, leading to significant economic losses and potentially causing safety accidents. Fault diagnosis methods for bearings can be divided into two categories: mechanistic model-based methods and data-driven methods. Mechanistic model-based approaches mainly build models based on mathematical and physical equations, which require a large amount of a priori knowledge and in-depth understanding, making it extremely difficult to establish an accurate mechanistic model. Data-driven methods include signal processing techniques, shallow machine learning methods, and deep learning methods. Signal processing methods mainly include wavelet transform (WT) [2,3], local mean decomposition (LMD) [4], empirical modal decomposition (EMD) [5], and short-time Fourier transform (STFT) [6,7]. The main shallow machine learning methods are support vector machine (SVM) [8,9], k-nearest neighbor (KNN) [10], and random forest (RF) [11]. Zheng et al. [12] implemented fault classification by calculating the composite multiscale fuzzy entropy (CMFE) of vibration signals based on ensemble support vector machine (ESVM). Huang et al. [13] analyzed a feature extraction method based on improved EMD energy entropy and used a genetic algorithm-based SVM as a classifier to classify faults. Yan et al. [14] proposed a new method to obtain feature vectors, instantaneous energy distribution-permutation entropy (IED-PE), by decomposing the vibration signal with improved VMD (IVMD) to obtain the components and then using KNN to achieve rolling bearing fault diagnosis.

However, traditional signal processing methods based on shallow machine learning struggle to meet the requirements of scalability, adaptability, and real-time performance in edge computing environments of the IIoT. These methods heavily rely on manual feature extraction, lack the ability to diagnose faults in complex high-dimensional signals, and are directly influenced by the expertise of personnel, making them unsuitable for different types of devices or operating conditions.

In recent years, deep learning models have gained increasing popularity in the field of fault diagnosis due to their efficient and reliable feature extraction capabilities. The convolutional neural network (CNN), as one of the most popular deep learning methods, can effectively extract deep features from data, making it highly applicable to fault diagnosis. Sun et al. [15] proposed a hybrid model based on CNN and long short-term memory (LSTM) networks, achieving higher fault diagnosis accuracy and robustness under different operating conditions. Zhang et al. [16] developed a CNN model incorporating an attention mechanism and dynamic learning rate for the intelligent diagnosis and identification of early faults in rolling bearings.

CNN has significant advantages over other deep learning methods for image processing and is specifically designed for processing 2D or 3D data. In some studies, raw data are converted into images to serve as input for CNNs. Ayas et al. [17] converted fault signals into grayscale images and used a deep residual network to learn the end-to-end mapping relationship between the images and the health condition of the moving bearings, enabling fault diagnosis. Xu et al. [18] transformed bearing vibration signals into time-frequency images using continuous wavelet transform and employed CNNs for fault feature extraction, which were then input into a classifier to achieve fault classification. Amarouayache et al. [19] utilized ensemble empirical modal decomposition (EEMD) to reconstruct the original signal and then converted it into a two-dimensional image for input into a CNN for fault classification. He et al. [20] used Markov transform fields to convert bearing vibration signals into images, combined with deep residual networks to extract deep information from the images for fault diagnosis. Wen et al. [21] first converted bearing vibration signals into grayscale maps and then used a modified LeNet-5 model for fault diagnosis. Yuan et al. [22] proposed a method based on the Hilbert–Huang transform combined with CNN and successfully applied it to the fault diagnosis of rolling bearings. Additionally, many CNN-based architectures, such as VGG-net [23] and Res-net [24], have been proposed as image feature extractors and classifiers and have also been applied to the fault diagnosis of image-type inputs.

Although the above methods achieve the function of fault diagnosis, there are still the following problems: (1). When preprocessing the sensor signal, the reconstruction method is not perfect, and it is difficult to effectively retain the time-dependent relationship of the original signal, which is easy to cause the loss of information. (2). Although CNN networks have excellent local feature capture capability, they do not capture enough global features. Using CNN to extract global features requires multi-layer stacking, and the amount of information decays as the number of layers increases.

Based on the above analysis, this paper proposes a novel method for rolling bearing fault diagnosis that combines Gramian Angular Field (GAF), Convolutional Neural Network (CNN), and Vision Transformer (ViT). The main contributions of this paper are summarized as follows.

1. Using GAF to Convert Signals: The GAF converts one-dimensional vibration signals into two-dimensional images, preserving the temporal correlation of the original vibration signals and retaining sufficient fault features. This overcomes the limitations in representing fault features of complex signals;
2. Parallel Architecture of CNN and ViT: The proposed model employs a parallel architecture of CNN and ViT for fault diagnosis. CNN collects local features hierarchically by cascading convolution operations and retains local clues as feature maps. Meanwhile, ViT aggregates global representations by cascading self-attention modules. This effectively combines the local feature extraction capability of CNN with the global feature extraction capability of ViT.

The integration of these techniques ensures high accuracy and robustness of the model when handling complex high-dimensional signals, thereby enhancing the abstraction and representation capabilities for complex signals. Ultimately, our method significantly improves the accuracy of bearing fault diagnosis by effectively representing the features of the original vibration signals and their potential relationships, and this has been validated on popular bearing datasets.

The rest of this paper is organized as follows. Section 2 introduces the related basic theory and our bearing fault diagnosis method. Section 3 describes experiments and results validation. In the end, the main conclusions are given in Section 4.

## 2. Methods

### 2.1. Gramian Angular Field

The Gramian Angular Field is a method for encoding a time series as a two-dimensional image. In the conventional Cartesian coordinate system, the time series is a one-dimensional signal where the x-axis represents the sampling points in time order, and the y-axis represents the amplitude of the sampled signal. The encoding process is as follows:

First, the time domain signal $X=\{x_{1},x_{2},x_{3},\ldots ,x_{n}\}$ in the Cartesian coordinate system is normalized to be between −1 and 1. Here, $x_{i}$ is the sampled points on the original time series, and ${\tilde{x}}_{i}$ represents the sampled points after normalization. The normalization process is shown in Equation (1).

(1) $${\tilde{x}}_{i}=\frac{x_{i}-\max X+x_{i}-\min X}{\max X-\min X}$$

Next, the normalized values are transformed into polar coordinates by encoding the values ${\tilde{x}}_{i}$ in ${\tilde{X}}_{i}$ as angular cosines ϕ and the timestamps as radii *r*. Thus, the time-domain signal *X* is transformed into a time series in polar coordinates, as shown in Equation (2).

(2) $$\left\{\begin{array}{c} \phi =\arccos{\tilde{x}}_{i};-1\leq {\tilde{x}}_{i}\leq 1,{\tilde{x}}_{i}\in \tilde{X}\\ r=\frac{t_{i}}{N};t_{i}\in N \end{array}\right.$$

where ${\tilde{X}}_{i}$ is the normalized time series, $t_{i}$ is the timestamp, and *N* is the constant factor of the generating space of the regularized polar coordinate system.

After converting the normalized time series into polar coordinates, the cosine value of the polar angle sum or polar angle difference between each time series point can be considered separately to identify the time correlation in different time intervals [25]. Therefore, it can be divided into the Gramian Angular Difference Field (GADF) based on the sine function and the Gramian Angular Sum Field (GASF) based on the cosine function, as defined by Equations (3) and (4).

(3) $$GADF=\left[\begin{array}{ccc} \cos\left(\phi_{1}-\phi_{1}\right) & \ldots & \cos\left(\phi_{1}-\phi_{n}\right)\\ \cos\left(\phi_{2}-\phi_{1}\right) & \ldots & \cos\left(\phi_{2}-\phi_{n}\right)\\ \vdots & \cos\left(\phi_{i}-\phi_{i}\right) & \vdots \\ \cos\left(\phi_{n}-\phi_{1}\right) & \ldots & \cos\left(\phi_{n}-\phi_{n}\right) \end{array}\right]$$

(4) $$GASF=\left[\begin{array}{ccc} \cos\left(\phi_{1}+\phi_{1}\right) & \ldots & \cos\left(\phi_{1}+\phi_{n}\right)\\ \cos\left(\phi_{2}+\phi_{1}\right) & \ldots & \cos\left(\phi_{2}+\phi_{n}\right)\\ \vdots & \cos\left(\phi_{i}+\phi_{i}\right) & \vdots \\ \cos\left(\phi_{n}+\phi_{1}\right) & \ldots & \cos\left(\phi_{n}+\phi_{n}\right) \end{array}\right]$$

After encoding, the temporal correlation of the 2D image from the top left to the bottom right is preserved, with the main diagonal containing the original information of the time series. Consequently, a time series of length *n* is encoded as a matrix of size *n* × *n*.

**Figure 1:**
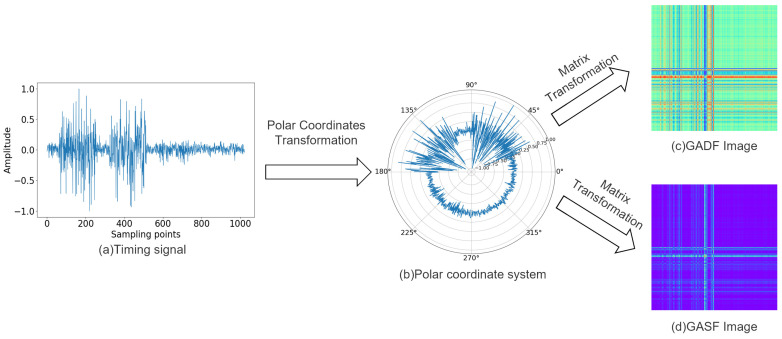
GAF encoding process.

The process of converting a one-dimensional signal into a two-dimensional image using GAF is illustrated in Figure 1. The GADF image exhibits richer colors compared to the GASF image, containing more detailed feature information.

The advantages of using the GAF method to encode time signals are as follows:

1. The 1D signal and 2D image are bi-mapped, and the polar coordinate transformation encoding preserves both the values and temporal positions of the points without any information loss. Thus, the transformation process retains the complete information of the 1D signal;
2. The time order of the signal is represented from the upper left corner to the lower right corner of the matrix, ensuring that the converted matrix retains the time dependence of the original time signal.

### 2.2. Vision Transformer

#### 2.2.1. Patch Embedding

For an image with an input size of *H* × *W* × *C*, the patch embedding operation is first to partition it into feature blocks of size *P* × *P*. The image is split into (H/P) × (W/P) feature blocks, each with the same number of channels. Each feature block is then linearly mapped to a one-dimensional vector of length *P* × *P* × *C*. Finally, these one-dimensional vectors are combined to produce the output of the patch embedding process.

#### 2.2.2. Transformer Encoder

**Figure 2:**
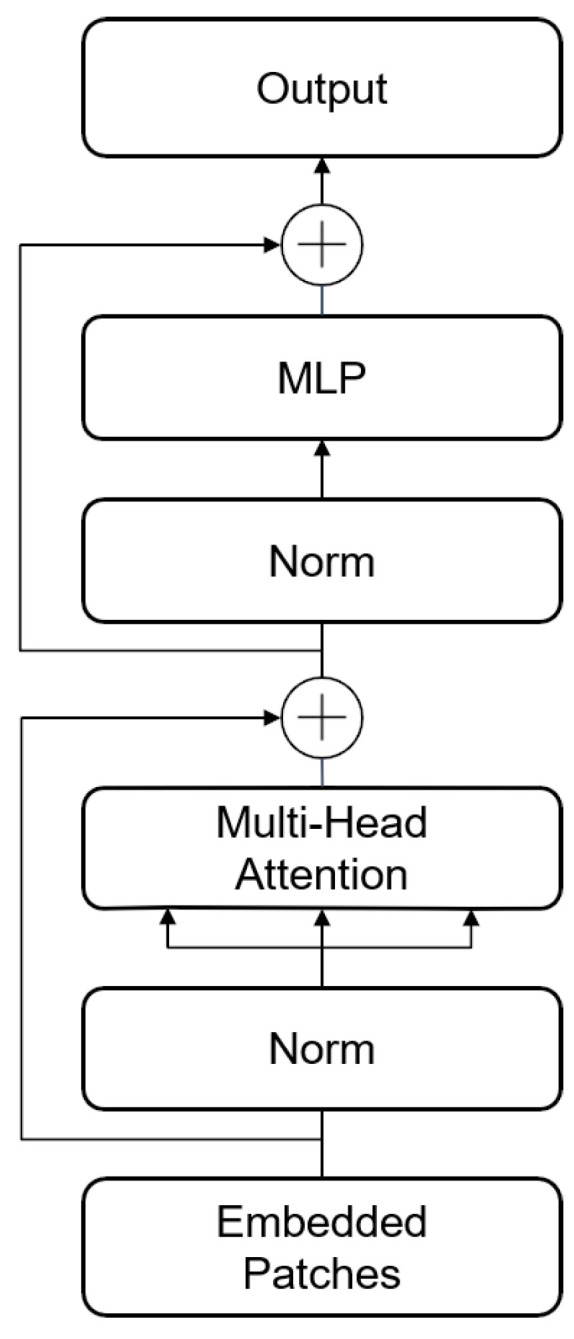
Transformer encoder.

The Transformer encoder, shown in Figure 2, includes a multi-headed self-attention (MSA) layer and a multi-layer perceptron (MLP) block. The computation process for the multi-headed self-attention layer used in this paper is as follows.

(5) $$Q=\mathrm{Linear}\left(X\right)=XW^{Q},\;Q_{i}=QW_{i}^{Q}$$

(6) $$K=\mathrm{Linear}\left(X\right)=XW^{K},\;K_{i}=KW_{i}^{K}$$

(7) $$V=\mathrm{Linear}\left(X\right)=XW^{V},\;V_{i}=VW_{i}^{V}$$

(8) $$\mathrm{Attention}\left(Q_{i},K_{i},V_{i}\right)=\mathrm{softmax}\left(\frac{Q_{i}K_{i}^{T}}{\sqrt{d_{K_{i}}}}\right)$$

(9) $${\mathrm{head}}_{i}=\mathrm{Attention}\left(Q_{i},K_{i},V_{i}\right)\times M+B$$

(10) $$\begin{array}{l} \mathrm{MultiHead}(Q,K,V)=\\ \mathrm{Concat}\left({\mathrm{head}}_{1},\ldots ,{\mathrm{head}}_{h}\right)\times W^{o} \end{array}$$

*Q*, *K*, and *V* are three matrices generated by the linear mapping of input *X* with different weights assigned. $\mathrm{Attention}\left(Q_{i},K_{i},V_{i}\right)$ is the computed inter-sequence association matrix of feature blocks. Based on the multi-headed self-attention layer of Vision Transformer, this paper proposes defining a set of learnable parameters, *M* and *B*. *M* is used to adaptively adjust the scale of the association matrix, while *B* adjusts the offset of the association matrix to enhance the model’s learning ability.

The MLP block comprises a fully connected layer, GELU activation function, and a Dropout layer with an inverted bottleneck structure. In this structure, the input feature layer is fully connected once, expanding the channel to four times its original size, and then the subsequent fully connected layer restores it to its original number. Layer normalization (LN) is applied before the residual connection in both the MSA and MLP blocks, which can be expressed by the following equation:

(11) $$X_{\mathrm{attention}}=X+\mathrm{MSA}\left(\mathrm{LN}\left(X\right)\right)$$

(12) $$\tilde{X}=X_{\mathrm{attention}}+\mathrm{MLP}\left(\mathrm{LN}\left(X_{\mathrm{attention}}\right)\right)$$

where *X* is the input sequence of feature blocks and $\tilde{X}$ is the output result.

#### 2.2.3. Position Embedding

Position embedding involves integrating a set of learnable parameters that signify the positional relationships of individual feature block sequences. The embedding of the position encoding can be expressed as follows in Equation (13).

(13) $$\tilde{x}=x+\theta$$

where *x* is the input feature block sequence and θ is a set of parameters for the learnable position.

### 2.3. Proposed Method

**Figure 3:**
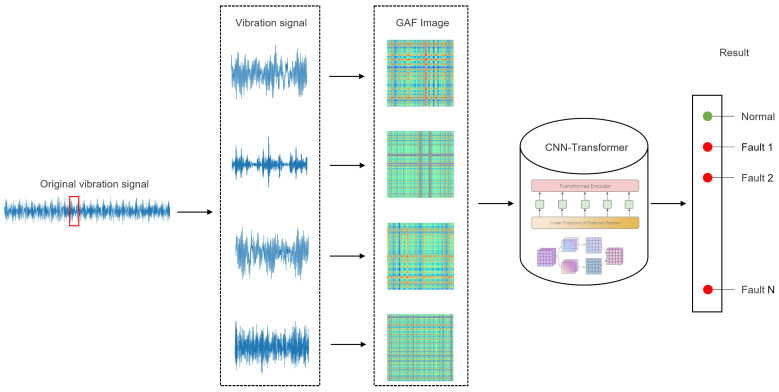
Fault diagnosis process based on GAF and CNN-ViT.

Incorporating principles from GAF, CNN, and Vision Transformer theories, we present a novel fault diagnosis approach that integrates feature extraction and fault pattern recognition, facilitating accurate and efficient fault classification. The fault diagnosis procedure is illustrated in Figure 3, with detailed steps outlined below.

1. Intercept samples of the original vibration signal in the dataset according to the well-defined length;
2. Utilize GAF to encode one-dimensional vibration signal samples into two-dimensional GAF images, thereby generating the image dataset;
3. The image dataset are input to the CNN-ViT model for training and testing;
4. Obtain the fault classification results.

#### 2.3.1. Signal-to-Image Conversion

The original vibration signal is initially captured as a series of samples. Subsequently, the one-dimensional vibration signal undergoes transformation into GADF and GASF matrices using the GAF method. These matrices serve as the basis for generating GADF and GASF images, wherein the elements within the matrices represent individual pixels. These images are then resized to a resolution of 224 × 224 with a three-channel configuration, resulting in an image size of 224 × 224 × 3. This resized image format is employed as the input for the CNN-ViT model.

#### 2.3.2. Construction of the Model

For GAF images, both global and local features are crucial. Traditional CNN models excel at extracting local information through convolution operations, capturing features at various levels, and producing feature maps via stacked convolutional layers. Conversely, ViT leverages self-attention modules to extract global image features starting from shallow layers [26]. To harness both global and local features effectively from GAF images, this paper introduces a model with a parallel architecture comprising both CNN and ViT components.

**Figure 4:**
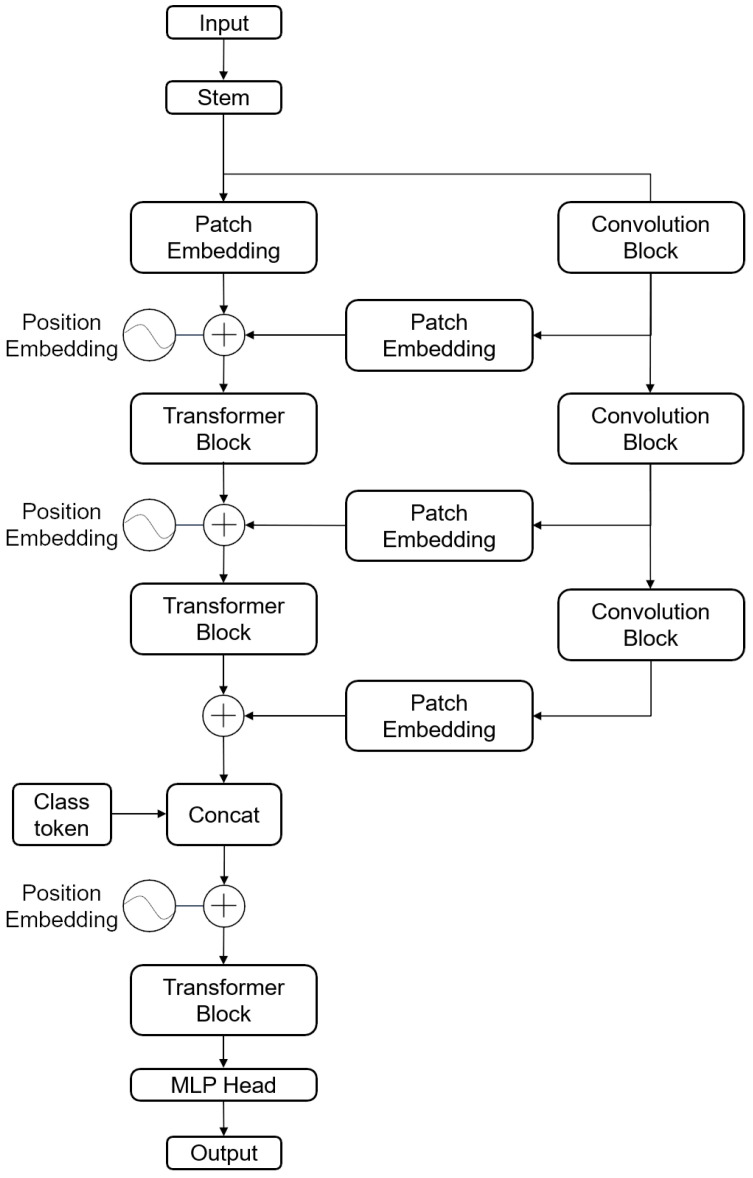
Structure of CNN-ViT model.

The architecture of the proposed CNN-ViT model, as shown in Figure 4, consists of three main parts: the stem block, the CNN branch, and the ViT branch. First, the GAF image is input into the stem block for initial feature extraction. This block captures multi-scale local features using three parallel paths with convolution and pooling operations of different kernel sizes (3 × 3, 5 × 5, and 7 × 7). The initially processed feature maps are then directed to both the CNN branch and the ViT branch. The CNN branch and the ViT branch each consist of multiple convolutional modules and Transformer modules. This parallel structure allows the model to maximally retain both local features and global representations. The Patch Embedding acts as a bridging module, linearly mapping the complete feature map into a sequence of feature patches and gradually transmitting the local feature map to the Transformer branch. This integration enables the local representation feature map from the CNN branch to merge with the global feature representation map from the Transformer branch. Additionally, positional encoding is added to understand the global context and long-range dependencies. Finally, the output results are obtained through a fully connected layer.

The CNN-ViT model extracts the fault features of GAF images in the following process.

**Figure 5:**
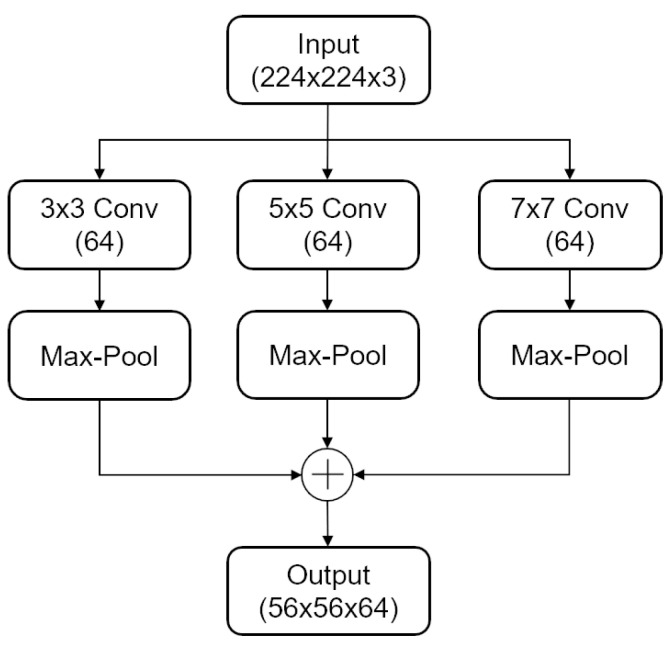
Stem block.

1. The stem block is used to extract initial local features, such as edge and texture information, from the GAF image using a three-branch parallel structure. In the first branch, a convolution operation of size 3 with a stride of 2 is applied, followed by a max pooling layer with a size of 3 and a stride of 2. The second branch involves a convolution operation of size 5 with a stride of 2, followed by a max pooling layer with a size of 3 and a stride of 2. Similarly, the third branch employs a convolution operation of size 7 with a stride of 2, followed by a max pooling layer with a size of 3 and a stride of 2. Local features at different scales of the original input image are extracted from each branch, and the resulting feature maps are aggregated by summation to serve as input for the CNN and ViT branches. For an input image of size 224 × 224 × 3, the output after the stem module is a 56 × 56 × 64 feature map. The structure of the stem block is depicted in Figure 5.

2. In order to reduce the parameters and decrease the amount of operations, patch embedding selects a feature block size of 4 × 4. The first patch embedding in the Transfomer branch of the model divides the 56 × 56 × 64 feature map into 196 feature blocks of size 4 × 4 × 64, which are then transformed into an embedding of size 196 × 1024. The remaining three patch embedding transform the 56 × 56 × 64 feature maps output from the three convolutional blocks into embeddings of size 196 × 1024, which are dimensionally consistent with the sequence of feature blocks on the Transfomer branch, so they can be directly passed to the Transfomer branch for fusion in a summation manner.

3. The CNN branch is composed of three consecutive convolutional blocks, each applying convolutions with a kernel size of 3 and a stride of 1. This configuration preserves the spatial dimensions between the input and output feature maps. As the network progresses through these layers—gradually expanding its receptive field—it extracts local features across diverse scales [27], prior to forwarding them to the ViT branch. This integration strategy counterbalances the potential lack of local feature extraction within the ViT branch, which directly transforms the feature map into a sequence of feature blocks via the Patch Embedding operation.

4. We add position encoding to the sequence of feature blocks. Following position encoding, the size, number, and the number of channels of the sequence of feature blocks remain unchanged.

5. After position encoding, the embedded data are fed into a Transformer block, which consists of multiple Transformer encoders stacked together. Specifically, there are 2, 2, and 6 of these encoders in each of the successive blocks, respectively. Prior to the final Transformer block, a category symbol is generated to represent the input feature block sequence, and this symbol is appended to the beginning of the original feature sequence. An MLP Head module follows, connecting a fully connected layer to this category symbol vector, with the ultimate aim of outputting the fault category.

6. The combination of local and global features is achieved through branch fusion. Each convolutional block in the CNN branch extracts local features from the image without changing the feature map size. After patch embedding, the dimensions are aligned with the global features in the ViT branch. Before being input to the subsequent processing layers, the feature vectors are summed, thereby aggregating the feature information of the entire image.

## 3. Experimental Validation

In this paper, we use CWRU and XJTU-SY bearing dataset to validate the effectiveness and practicality of the GAF combined with CNN-ViT fault diagnosis method. The experimental platform is Python3.7 + Pytorch1.7.1, and the hardware is Intel Xeon(R) Gold 6226R + NVIDIA GeForce RTX 3090.

### 3.1. Data Description

The Case Western Reserve University (CWRU) dataset was provided by the Case Western Reserve University Bearing Data Center [28]. Vibration signals from the experimental platform, obtained from both normal and defect-bearing conditions, were recorded at frequencies of 12 kHz or 48 kHz under four distinct motor load scenarios. Under each operational condition, faults characterized by diameters of 0.007, 0.014, and 0.021 inches were artificially induced in the rolling elements, inner rings, and outer rings, respectively. This study employs data sampled at 12 kHz from the drive end, categorizing them into one healthy state and three failure modes: inner ring failure, rolling element failure, and outer ring failure. Further, these conditions are stratified into ten classes based on the extent of failure: comprising one healthy state and nine distinct failure states. Table 1 outlines the fault classifications alongside their respective labels for the CWRU dataset.

XJTU-SY bearing dataset were provided by the Institute of Design Science and Basic Component at Xi’an Jiaotong University and the Changxing Sumyoung Technology Co., Ltd. (Huzhou, China) [29,30]. In this paper, experiments were conducted using Bearing1 from the dataset with a sampling frequency of 2.56 kHz. Five categories were classified according to the actual lifetime as well as the failure location, and each failure state was uniquely thermally coded. Table 2 describes the failure categories and corresponding labels of XJTU-SY dataset.

### 3.2. Data Processing

Given the limited length of the bearing vibration signal for each fault condition, we employ sliding window sampling to increase the sample size. The sliding window progresses with a step equivalent to its movement distance, set at a window length of 1024 samples and a step size of 512 samples, thereby sampling at a 50% overlap rate. This approach doubles the sample count while mitigating feature loss that would otherwise result from direct truncation of samples [31].

**Figure 6:**
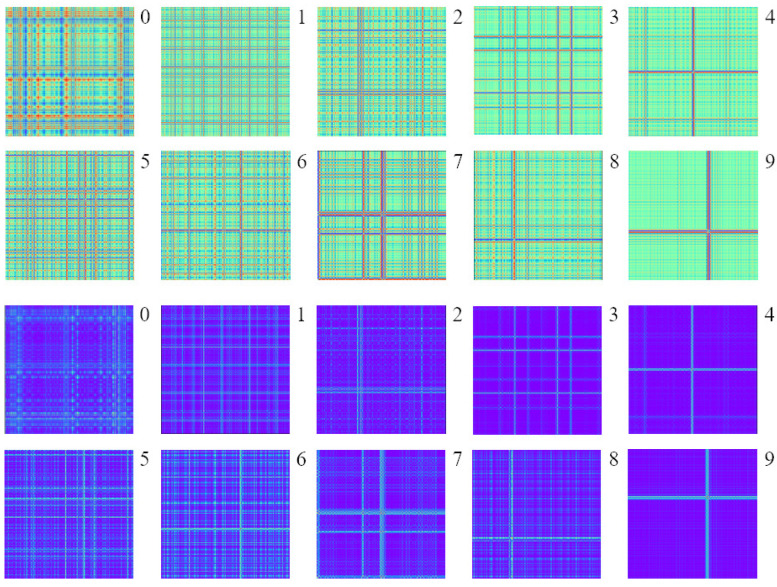
Ten GAF images based on CWRU bearing dataset. The green ones are GADF images and the purple ones are GASF images.

**Figure 7:**
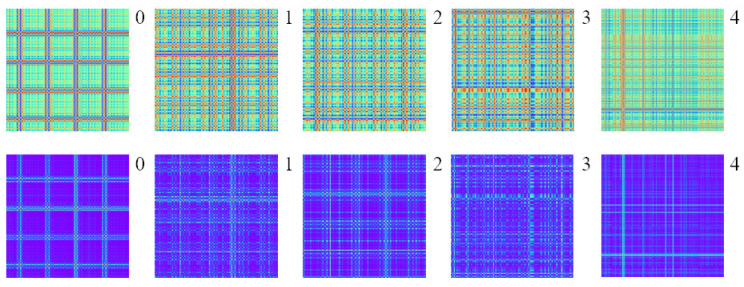
Five GAF images based on XJTU-SY bearing dataset. The green ones are GADF images and the purple ones are GASF images.

Each category of failure state signals of CWRU bearing data is divided into 2400 samples by sliding window sampling, i.e., 240 samples for each category. Then, the vibration signal samples are converted into 2400 images by GAF coding as the data set, and the GAF images of ten fault states are shown in Figure 6. Similarly, each type of fault state signal of XJTU-SY bearing data is divided into 4000 samples by sliding window sampling, i.e., 800 samples for each category, and then, the vibration signal samples are converted into 4000 images by GAF coding as the data set, and the GAF images of five fault states are shown in Figure 7.

### 3.3. Experimental Results

In order to avoid classification bias caused by specific data splits, 80% of the dataset was randomly selected as the training set and the remaining 20% as the test set, for a total of 10 training rounds. The model parameters are as follows: learning rate is 0.0001, batch size is 64, number of epochs is 200, optimizer is Adam, and the loss function is cross-entropy.

**Figure 8:**
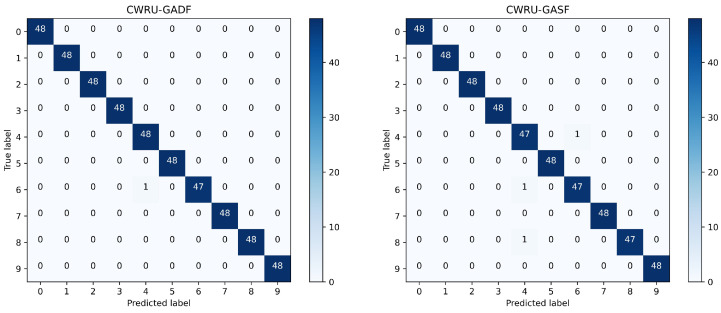
Confusion matrix of CWRU classification results.

**Figure 9:**
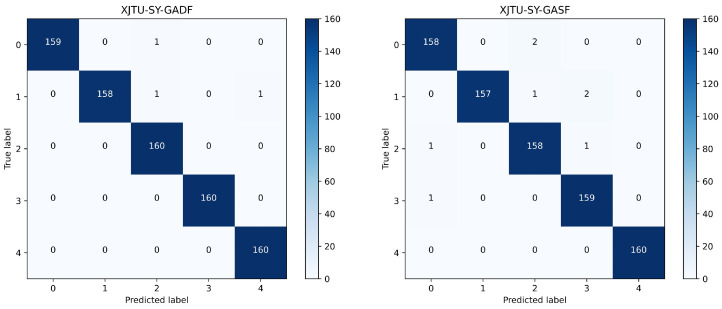
Confusion matrix of XJTU-SY classification results.

To better show the experimental results of the model for different fault types, a confusion matrix was introduced to visualize the prediction results, where the horizontal axis is the prediction label, and the vertical axis is the true label. The confusion matrix of the classification results for ten bearing states of the CWRU dataset is shown in Figure 8, and the confusion matrix of the classification results for five bearing states of the XJTU-SY dataset is shown in Figure 9.

We conducted several experiments, and the average recognition rates of GADF and GASF images in the test set were 99.79% and 99.38%, respectively, in the CWRU dataset, and 99.63% and 99.00%, respectively, in the XJTU-SY dataset. In the experiments conducted using the CWRU dataset, we found that the method proposed in this paper accurately and clearly predicts faults or non-faults, with very few fault-free images being predicted as faulty. Additionally, nearly all misclassifications occurred in ball faults, with undetected inner or outer ring faults possibly caused by Brinell hardness. In the experiments using the XJTU-SY dataset, we found that the proposed method generally accurately identifies the specific location of rolling bearing faults, but it tends to misclassify different fault states at the same location.

**Figure 10:**
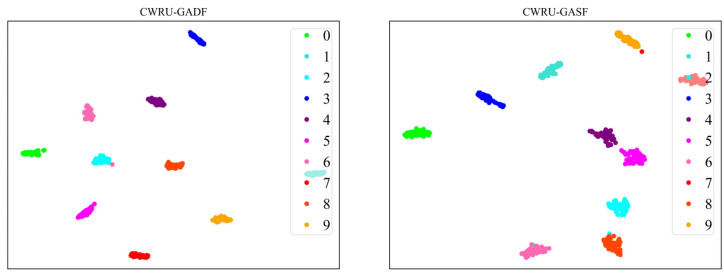
t-SNE visualization of GAF images fault feature extraction results for CWRU dataset.

**Figure 11:**
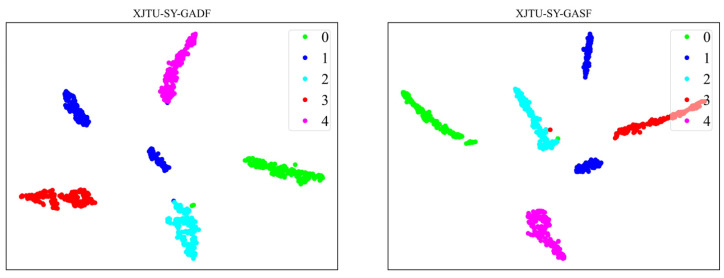
t-SNE visualization of GAF images fault feature extraction results for XJTU-SY dataset.

To better illustrate the performance of the model, we employ the nonlinear dimensionality reduction algorithm, t-SNE [32], to project and visualize the data in a two-dimensional space. Specifically, for the CWRU dataset, Figure 10 showcases the t-SNE visualization results of the CNN-ViT model when extracting fault features from both GADF and GASF images. Meanwhile, the t-SNE visualization outcomes for the XJTU-SY dataset, illustrating the CNN-ViT model’s feature extraction from GADF and GASF images, are depicted in Figure 11.

**Figure 12:**
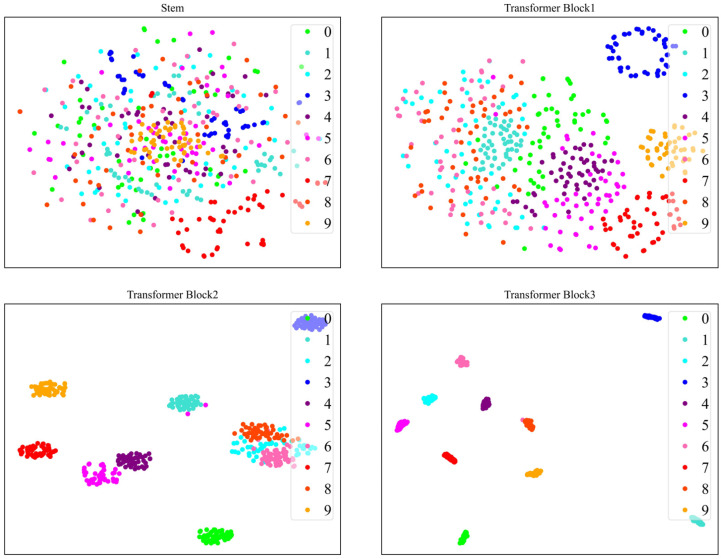
t-SNE visualization of feature extraction process for GADF images of CWRU dataset.

**Figure 13:**
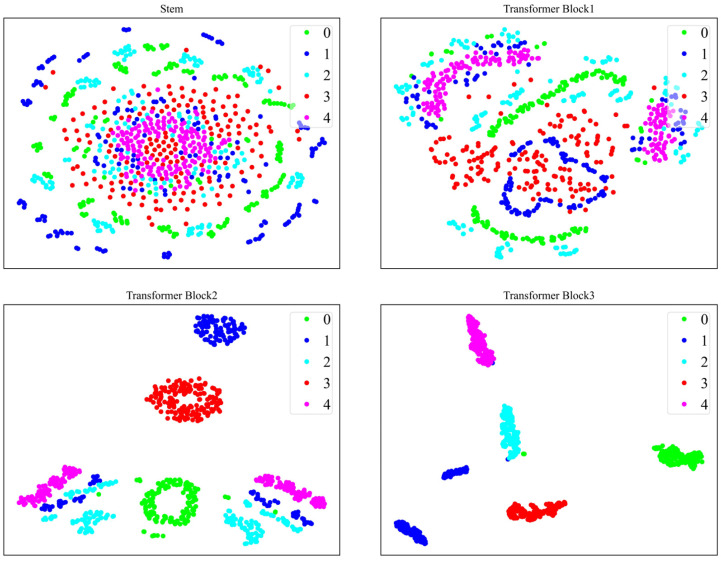
t-SNE visualization of feature extraction process for GADF images of XJTU-SY dataset.

In order to understand the process of feature extraction by the model more clearly, we visualize the outputs from the Stem module and the three Transformer Blocks using t-SNE. Taking the GADF images from both the CWRU and XJTU-SY datasets as examples, Figure 12 and Figure 13, respectively, illustrate this process.

Initially, the Stem module extracts initial local features from the raw GADF image; however, at this stage, distinguishing fault samples belonging to different categories is difficult. As the process progresses through the CNN branch and ViT branch, which incrementally extract deeper image features, the sample distribution evolves, with faults of the same category beginning to cluster together. Ultimately, following the comprehensive feature extraction by the CNN-ViT model, a superior separation of sample features across different fault categories is achieved, highlighting the effectiveness of the model’s feature extraction capabilities.

**Figure 14:**
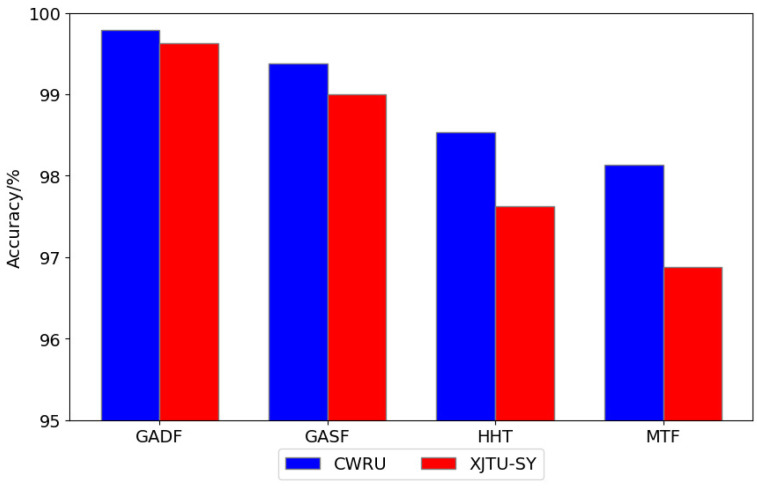
Fault diagnosis test results of different image coding methods.

From the above experiments, it can be seen that the CNN-ViT architecture proposed in this paper achieves better fault diagnosis results on public datasets by introducing convolution operators from CNNs to extract local features of images and self-attention mechanisms from Transformers to capture global representations. To further verify the reliability of the image encoding method proposed in this paper, we compared it with other image encoding methods. One method converts the original one-dimensional signal into a time-frequency map based on the Hilbert–Huang Transform (HHT), and another method encodes the original one-dimensional signal into feature images based on the Markov Transition Field (MTF). The training and testing sets were divided in the same proportion. The training and testing sets were then input into the CNN-ViT model for feature extraction and fault classification. The results, as shown in Figure 14, indicate that the recognition rates of GAF images are higher than those of HHT images and MTF images, with the highest recognition rate achieved by GADF images. This demonstrates that the GADF image encoding method has better signal reconstruction ability and can more effectively express vibration signal characteristics, making it more suitable for bearing fault classification and diagnosis compared to other image encoding methods.

To verify the advantages of the CNN-ViT model in bearing fault diagnosis, comparative experiments were conducted with various fault classification models, and the results are presented in Table 3 and Table 4. The LSTM and SVM algorithms, which rely on one-dimensional vibration signals as input, struggle to meet the rigorous demands of high-precision fault diagnosis, with their diagnostic accuracy generally trailing behind that of deep learning models employing GAF-encoded images of these one-dimensional signals. This evidences that encoding vibration signals into GAF images markedly enhances the overall recognition accuracy of the model. Furthermore, in order to comprehensively evaluate the performance, the study incorporated the VGG16 model, embodying a classic CNN architecture, and the ViT model for comparative experiments. Whether tested on the CWRU dataset or the XJTU-SY dataset, the CNN-ViT model proposed here achieved slightly higher classification accuracy compared to instances where the CNN and ViT architectures were used independently under the same input conditions. These results demonstrate that the proposed CNN-ViT model has strong feature learning and fault diagnosis capabilities, allowing it to accurately identify bearing status.

## 4. Conclusions

In this paper, a fault diagnosis method that can be used for edge devices is proposed. The one-dimensional vibration signal is encoded into a two-dimensional image by GAF, the original signal from the sensor is reconstructed, and the time correlation is retained. A CNN-ViT model for rolling bearing condition identification was constructed. Experimental results on CWRU and XJTU-SY bearing datasets show that the proposed fault diagnosis method achieves average fault recognition accuracies of 99.79% and 99.63%, respectively. Compared to other intelligent bearing fault diagnosis methods, it exhibits better feature extraction and classification performance, providing a reliable solution for fault diagnosis on edge devices. However, this study has certain limitations. Firstly, the evaluation of the model is focused on the CWRU and XJTU-SY datasets, which implies a dependence on specific datasets. Therefore, future research should consider validation on a more diverse range of industrial datasets to ensure the general applicability of the research findings. The fault diagnosis method proposed in this paper involves many hyperparameters, most of which are set based on experimental experience, posing the risk of overfitting to the training data and compromising the model’s generalization to unseen instances. In future work, we will explore intelligent hyperparameter tuning algorithms, including Bayesian optimization or neural architecture search, to systematically explore the vast hyperparameter space. This will not only mitigate the potential risk of overfitting but also accelerate the model development cycle, making it more suitable for deployment on resource-constrained edge devices.

## Figures and Tables

**Table 1:** Description of CWRU sample categories.

Fault Location	Fault Diameter (Inch)	Label
NO	0	0
Inner ring	0.007	1
Inner ring	0.014	2
Inner ring	0.021	3
Rolling Element	0.007	4
Rolling Element	0.014	5
Rolling Element	0.021	6
Outer ring	0.007	7
Outer ring	0.014	8
Outer ring	0.021	9

**Table 2:** Description of XJTU-SY sample categories.

File	Lifetime	Fault Element	Label
Bearing 1_1	2 h 3 min	Outer ring	0
Bearing 1_2	2 h 41 min	Outer ring	1
Bearing 1_3	2 h 38 min	Outer ring	2
Bearing 1_4	2 h 2 min	Cage	3
Bearing 1_5	52 min	Inner ring and Outer ring	4

**Table 3:** Experimental results of CWRU dataset.

Method	Data Type	Accuracy
CNN	GADF	99.58%
CNN	GASF	98.96%
Vision Transformer	GADF	99.17%
Vision Transformer	GASF	98.54%
CNN-ViT	GADF	99.79%
CNN-ViT	GASF	99.38%
LSTM	Original signal	95.83%
SVM	Original signal	96.45%

**Table 4:** Experimental results of XJTU-SY datasett.

Method	Data Type	Accuracy
CNN	GADF	99.13%
CNN	GASF	98.50%
Vision Transformer	GADF	99.00%
Vision Transformer	GASF	98.25%
CNN-ViT	GADF	99.63%
CNN-ViT	GASF	99.00%
LSTM	Original signal	94.75%
SVM	Original signal	95.50%

## Data Availability

The data are publicly available.
